# Post-transcriptional regulation in the *myo1Δ *mutant of *Saccharomyces cerevisiae*

**DOI:** 10.1186/1471-2164-11-690

**Published:** 2010-12-02

**Authors:** Marielis E Rivera-Ruiz, José F Rodríguez-Quiñones, Pearl Akamine, José R Rodríguez-Medina

**Affiliations:** 1 Department of Biochemistry, School of Medicine, Medical Sciences Campus, University of Puerto Rico, P.O. Box 365067, San Juan, PR 00936-5067, U.S.A; 2 Department of Medicine, Center for Aging, Tulane University, New Orleans, LA, U.S.A

## Abstract

**Background:**

*Saccharomyces cerevisiae *myosin type II-deficient (*myo1*Δ) strains remain viable and divide, despite the absence of a cytokinetic ring, by activation of the *PKC1*-dependent cell wall integrity pathway (CWIP). Since the *myo1*Δ transcriptional fingerprint is a subset of the CWIP fingerprint, the *myo1*Δ strain may provide a simplified paradigm for cell wall stress survival.

**Results:**

To explore the post-transcriptional regulation of the *myo1*Δ stress response, 1,301 differentially regulated ribosome-bound mRNAs were identified by microarray analysis of which 204 were co-regulated by transcription and translation. Four categories of mRNA were significantly affected - *protein biosynthesis, metabolism, carbohydrate metabolism*, and *unknown functions*. Nine genes of the 20 CWIP fingerprint genes were post-transcriptionally regulated. Down and up regulation of selected ribosomal protein and cell wall biosynthesis mRNAs was validated by their distribution in polysomes from wild type and *myo1Δ *strains. Western blot analysis revealed accumulation of the phosphorylated form of eukaryotic translation initiation factor 2 (eIF2α-P) and a reduction in the steady state levels of the translation initiation factor eIF4Gp in *myo1Δ *strains. Deletion of *GCN2 *in *myo1Δ *abolished eIF2αp phosphorylation, and showed a severe growth defect. The presence of P-bodies in *myo1Δ *strains suggests that the process of mRNA sequestration is active, however, the three representative down regulated RP mRNAs, *RPS8A, RPL3 *and *RPL7B *were present at equivalent levels in Dcp2p-mCh-positive immunoprecipitated fractions from *myo1Δ *and wild type cells. These same RP mRNAs were also selectively co-precipitated with eIF2α-P in *myo1Δ *strains.

**Conclusions:**

Quantitative analysis of ribosome-associated mRNAs and their polyribosome distributions suggests selective regulation of mRNA translation efficiency in *myo1*Δ strains. Inhibition of translation initiation factor eIF2α (eIF2α-P) in these strains was by Gcn2p-dependent phosphorylation. The increase in the levels of eIF2α-P; the genetic interaction between *GCN2 *and *MYO1*; and the reduced levels of eIF4Gp suggest that other signaling pathways, in addition to the CWIP, may be important for *myo1*Δ strain survival. Selective co-immunoprecipitation of RP mRNAs with eIF2α-P in *myo1*Δ strains suggests a novel mode of translational regulation. These results indicate that post-transcriptional control is important in the *myo1*Δ stress response and possibly other stresses in yeast.

## Background

Controlled regulation of gene expression is essential for maintaining the normal metabolic functions in living cells as well as for producing changes in cellular functions in response to life-threatening environmental and physiological stress. In yeast, the *PKC1 *cell wall integrity pathway (CWIP) represents one of the primary signaling pathways regulating gene expression in response to stress such as cell wall damage, heat shock, and disruption of polarized growth [1-4]. Its activation is marked by the accumulation of the hyperphosphorylated MAP kinase, Slt2p/Mpk1p. A gene transcription fingerprint for cell wall damage, which includes Slt2p, consists of 20 genes of different biological functions previously described by others [2,5].

The *Saccharomyces cerevisiae *myosin type II heavy chain (Myo1p) is a protein required for normal cytokinesis in yeast. It was previously shown that hyperphosphorylated Slt2p accumulates in the deletion mutant of Myo1p (*myo1Δ*) [6] as well as in *chs2*Δ [7], a related cytokinesis mutant strain in which chitin synthase 2, a protein important for primary septum formation, has been deleted [8]. Furthermore, the *slt2Δ *mutation was lethal in *myo1Δ *suggesting that its expression was required for survival [6]. However, when we examined the *myo1Δ *and *chs2Δ *strains for expression of the cell wall damage fingerprint, we found that despite morphological and biochemical phenotypes shared between *myo1Δ *and *chs2Δ *cytokinesis mutants, they expressed distinctively different cell wall damage fingerprints setting *myo1Δ *apart from the *chs2Δ *[7]. The *myo1Δ *strain therefore appears to have a unique mode of survival in response to stress and thus is an alternative model system for impaired cytokinesis and cell wall stress.

To further characterize how *myo1Δ *mounts its unique stress response, we previously described the global mRNA expression profile using microarray hybridization [6]. Knowing that the steady state levels of mRNA transcripts are a product of transcription rates and decay rates, and that mRNA levels are regulated at both the transcriptional and/or the post-transcriptional levels, we chose to study global gene regulation at these different levels to fully understand the complexity of genetic interactions that occur in response to the stress caused by the *myo1Δ *mutation.

Transcriptional regulation is traditionally the most studied because it represents the first level of regulation of genes and because technically speaking, it can be probed by widely accessible genome-wide mRNA expression profiling techniques [2,5-7,9,10]. Post-transcriptional regulation including translation control and mRNA decay is also used by cells to modulate gene expression in a wide range of biological situations and is critical under conditions that require sudden and precise changes in protein levels including the cellular response to stress and apoptosis [11-15]. For example, a global inhibition of protein synthesis has been reported under different types of stress including: entry into diauxic shift [13], ethanol exposure [14], oxidative [15] and osmotic stress [16]. Inhibition of protein synthesis prevents continued gene expression during potentially error-prone conditions and may accelerate the turnover of existing mRNAs [15]. In yeast, translational regulation occurs by several mechanisms, including decreased synthesis of rRNA, reduction in steady state levels of the translation initiation complex eIF4F (composed of eIF4Ep, eIF4Gp, and eIF4Ap) [17], and phosphorylation of the alpha subunit of the initiation factor eIF2 (eIF2αp, encoded by *SUI2*) [18,19].

Yeast cells mount changes in gene expression that are coordinated with changes in the translatome in their response to different types of environmental stresses [9,10]. This co-regulation of genes by transcription and translation has been demonstrated for genes that fall within functional categories of carbohydrate metabolism and energy production and is believed to be important for activating the environmental stress response (ESR) [10]. The extent of co-regulation can be correlated to the severity of the stress involved. Therefore, we examined both transcriptional and post-transcriptional mechanisms and their coordination in order to uncover potentially important stress response genes.

Cytoplasmic processing bodies or P-bodies are ribonucleoprotein aggregates that have been implicated in storage of mRNAs, translation repression, and mRNA degradation [20], another means of post-transcriptional regulation. P-bodies exist in yeast with similar function and protein composition to P-bodies in mammalian cells. For different stresses, a decrease in mRNA translation rate correlates with an increase in the formation of P-bodies, where non-translating mRNAs can be sequestered into these structures [20]. Dcp2p is a protein of the P-bodies found in yeast cells and has been used as a marker for *in vivo *localization studies [20,21]. Therefore, we analyzed mRNA composition of Dcp2p-mCh-positive immunoprecipitated protein fractions in our *myo1Δ *strain to determine if these fractions were involved in the post-transcriptional regulation of the differentially expressed genes.

Here, we studied the relative abundance of translationally regulated genes by microarrays analysis. The regulated genes were then classified according to their biological functions. This analysis reiterated the importance of protein biosynthesis down regulation found before [6] and showed a significant number of ribosomal protein (RP) genes that were down regulated at the transcriptional level were also translationally repressed in the *myo1Δ *mutant. The polyribosome distribution of representative RP mRNAs supported the results from the DNA microarrays. The *myo1Δ *cells down regulated translation by Gcn2p-dependent phosphorylation of eIF2αp and reduced steady state levels of the translation initiation factor eIF4Gp, suggesting that the *TOR *pathway may be involved in the *myo1Δ *stress response in addition to the *PKC1 *cell wall integrity pathway. The conclusion of this study is that post-transcriptional regulation of gene expression is an important mechanism for cell survival under conditions of impaired cytokinesis and cell wall stress in the *myo1Δ *mutant.

## Methods

### Strains and Growth conditions

Experiments were performed using *Saccharomyces cerevisiae *wild type, *myo1*Δ, *gcn2*Δ, and *myo1*Δ*gcn2*Δ, strains (Table 1). Cultures were grown overnight at 26°C between 0.5-0.8 OD_600 _in Complete Synthetic Media (CSM) supplemented with 2% glucose and 1× Nitrogen base, with continuous shaking at 200 rpm. Dr. Brian C. Rymond kindly provided a *GCN2 *null mutation strain. A *myo1Δgcn2Δ *double mutant was constructed by replacing the *MYO1 *gene with a *HIS5 *module by homologous recombination using a PCR- based method. This strain was transformed with a plasmid copy of *MYO1^+ ^(pRS316-MYO1^+^) *to test for rescue of the double mutant phenotype. Colonies were confirmed by PCR analysis. For visualization of P-bodies structures by fluorescence microscopy, wild type and *myo1*Δ strains were transformed using a Dcp2p-mCh (p1658) plasmid (kindly provided by Dr. Roy Parker) by the LiOAC method [22]. Microscopic examination was performed as previously described [23]. Briefly, live transformed cells were wet-mounted on a glass slide with cover slip, illuminated with U.V. light using a Texas Red filter with 596 nm excitation and 620 nm emission wavelengths (Olympus America, Inc., Melville, NY). Images were observed at 100× magnification and photographed with a Spot RT-cooled CCD camera (Diagnostic Instruments Inc., Sterling Heights, MI). For the quantification of P-bodies by fluorescence microscopy Dcp2p-mCh-positive structures were counted in a minimum total of 100 cells. The percent of P-bodies was averaged from 3 independent experiments using the following formula: (number of P-bodies/number of cells counted) × 100.

**Table 1:** Strains used in this study.

Strain	Genotype	Source
YJR12 (wild type, wt)	MAT α trp1 ura3 leu2-3 his3delta1 ADE+ ARG+ cyh^R^	Lab Strain
YJR13 (*myo1Δ*)	MAT a trp1 ura3 leu2-3 his3delta1 ADE+ ARG cyh^R ^myo1delta::HIS5+	Lab Strain
*gcn2Δ*	MAT α his3delta1 leu2delta0 met15delta0 ura3delta0 gcn2delta::kanMX4	B. Rymond
*myo1Δgcn2Δ*	MAT α his3delta1 leu2delta0 met15delta0 ura3delta0 gcn2delta::kanMX4 myo1delta::HIS5+	This study
YJR12 p1658 *(pDCP2-mCh)*	MAT α trp1 ura3 leu2-3 his3delta1 ADE+ ARG+ cyh^R ^pDcp2p-mCh	This study
YJR13 p1658 *(myo1Δ pDCP2-mCh)* *myo1Δgcn2ΔpRS316-MYO1^+^*	MAT a trp1 ura3 leu2-3 his3delta1 ADE+ ARG cyhRmyo1delta::HIS5+ pDcp2p-mCh MAT α his3delta1 leu2delta0 met15delta0 ura3delta0 gcn2delta::kanMX4 myo1delta::HIS5+ pRS316-MYO1+	This study This study

### Viability assays

Viability assays were performed using 1 × 10 ^7 ^cell/ml aliquots taken from cultures at OD_600 nm _between 0.5-0.8. For agar plate assays, 5 μl from 1/10 serial dilutions (ranging from 10^7 ^to 10^3 ^cell/ml) were inoculated in CSM agar plates. Plates were incubated at 27°C for three days. Growth curves were also performed to validate these observations by inoculating 1 × 10 ^7 ^cell/ml aliquots in CSM media. OD_600 nm _measurements were taken every two hours for up to eight hours.

### Ribosome fractionation by differential centrifugation

Yeast cells were treated with 50 μg/ml of cycloheximide to halt translation immediately before harvesting by centrifugation (2,061 × g for 5 min at 4°C). Cell pellets were washed with ice-cold lysis buffer (50 mM Tris-HCl, pH 7.5, 100 mM NaCl, 7 mM MgCl_2, _1 mM DTT, 1 mM PMSF and 50 μg/ml cycloheximide) and then resuspended in 1.5 ml of lysis buffer together with a quarter volume of acid-washed 0.5 mm glass beads. Cells were disrupted by vortex mixing for 20 seconds with 30-second intervals on ice (repeated 10 times). Unbroken cells and large debris were removed by a low speed spin (800 × g for 10 min at 4°C). The supernatant was centrifuged at 10,000 × g for 30 min at 4°C, yielding supernatant (S10) and pellet (P10). Supernatant S10 was collected, layered onto an equal volume of a 50% sucrose solution and centrifuged at 100,000 × g for 60 min at 4°C (Beckman 80 Ti rotor) thereby yielding supernatant S100 and ribosomal pellet P100. A novel method has been described recently for affinity purification of ribosomes [10]. This method allows purification of polysomes that are devoid of non-ribosomal cellular components which may co-purify with ribosomes by the classical 50% sucrose cushion method used here and can increase recovery of free ribosomes and/or lighter polysomes that may remain in the S100 fraction [[10], and our unpublished observations].

### RNA Extraction

Ribosomal pellets contained in P100 fractions, immunoprecipitation eluates, and sucrose density gradient fractions, were all resuspended in a guanidine thiocyanate buffer containing 10% mercaptoethanol (RLT buffer, RNeasy Mini Kit, Qiagen). RNA was extracted using the RNeasy Mini Kit for isolation of total RNA (Qiagen, Valencia, CA) following the manufacturer's instructions. RNA concentrations were determined by measuring absorbance at 260 nm using a Nanodrop spectrophotometer (Nanodrop Technologies, Wilmington, DE). The purity and integrity of the RNA was monitored by electrophoresis using an Agilent Bioanalyzer (Agilent Technologies, Palo Alto, CA) following the manufacturer's instructions. Typically we obtained approximately 20 μg of RNA from ribosome pellets and 0.1 μg of RNA from immunoprecipitation experiments. A control for DNA contamination (PCR of the RNA without a prior RT reaction) was routinely performed for each total RNA preparation.

### Yeast oligonucleotide microarray hybridization and analysis

Microarray hybridization experiments were each conducted with three biological and one technical replicates using total RNA extracted from ribosomal pellets of wild type and *myo1Δ *strains. Experiments were performed as described in [6], 1.0 μg of total RNA extracted from ribosomal pellets was amplified using the Low RNA Input Fluorescent Linear Amplification kit (Agilent Technologies, Palo Alto, CA). Amplified cRNA was labeled with 10 mM Cyanine 5-CTP (Cy5) or Cyanine 3-CTP (Cy3) (Perkin Elmer Life Sciences, Boston, MA). Labeled cRNAs were purified with Qiagen RNeasy mini spin columns and dye incorporation was monitored on an Agilent Bioanalyzer. Hybridization of Cy5 and Cy3 labeled cRNAs were performed using Yeast Oligo Microarrays slides and hybridization kit from Agilent Technologies (Sheldon Manufacturing, Cornelius, Oregon) at 60°C overnight. Slides were washed and scanned with a VersArray Chip Reader system (Bio-Rad, Hercules, CA) at a resolution of 5 μm with detector sensitivity values set between 704-800 and laser power at 85%. Scanned images were transferred to the Imagene 3.0 software program (Biodiscovery, El Segundo, CA) for further analysis to locate spots, adjust the appropriate grid, and obtain the Cy3 and Cy5 TIFF files. The microarrays raw data was then analyzed using Limma software (Bioconductor Package 1.7) [24]. The individual data sets were normalized using the locally weighted linear regression (Lowess) within each array. After normalization, the difference between the experimental and control signal was calculated, replicates were combined, and their averages were calculated. The fold change in gene expression was calculated by 2^(M)^, where M is the log_2_-fold change ratio after background correction and normalization. An Empirical Bayes Statistics for differential expression analysis (eBayes statistics) was performed [25]. A p-value ≤ 0.01 was established as the cutoff for differential expression. In addition, a false discovery rate (FDR) was performed with Limma software [26]. Microarray raw and processed data are available at the Gene Expression Omnibus (GEO) site of NCBI GSE20203 http://www.ncbi.nlm.nih.gov/geo/

### Gene Set Enrichment Analysis

Gene Set Enrichment Analysis was performed using the Limma package of Bioconductor [27]. Briefly, a gene set file was created classifying all the genes included in the microarray into groups according to their specific involvement in a biological process. From this gene set, a total of 25 categories were represented according to the Osprey network visualization software [Additional file 1] [28], matching the category with the t-value. Then, the Limma software calculated the average of the t-values for each biological process category to determine the average fold-change for the category. The significance of differential expression was determined through an enrichment analysis by calculating the significance after 10,000 permutations. The initial cutoff to determine that the gene set was differentially expressed was p≤ 0.01. The final corrected p-value obtained using the Bonferroni correction was p≤ 0.00056.

### Confirmation of microarray data by real time RT-PCR

Microarray results were corroborated by real time RT-PCR analysis using a subset of genes involved in cell wall and ribosome biogenesis. Real Time RT-PCR was performed with 25 ng of RNA extracted from ribosomal pellets using Quantitec SYBR Green RT-PCR kit (Qiagen, Valencia, CA) with primers at 0.5 μM (Table 2) and a final reaction volume of 25 μl. The reactions were performed following the conditions recommended by the manufacturer using the iCycler iQ Multicolor real time PCR detection system (Bio-Rad, Hercules, CA). The PCR quantification and melting curves were generated using the iCycler software (Bio-Rad, Hercules, CA). The fold change was determined by the 2^ΔΔCt ^method [29]. The ΔC_t _of the control and experimental samples was calculated from the threshold cycle of the target gene minus the threshold cycle of the reference gene (*ACT1*) in triplicate experiments. The ΔΔC_t _was then calculated by subtracting the averaged ΔC_t _of the control samples minus the averaged ΔC_t _of the experimental sample. Reagent controls were included to test for DNA contamination.

**Table 2:** Primers used in this study.

Gene	Forward Primer	Reverse Primer
*RPL3*	5'-GACGGTGCTGGTATTGAAAG-3'	5'-CCAGTCAACCTTTTCAGAGA-3'
*RPL7B*	5'-ACCAAGGCTACTTTGGAACT-3'	5'-GCTTCGATGATAGCATTGTC-3'
*RPS8A*	5'-GGTAACTTTTCTTGGGCTTC-3'	5'-GAACCATTGTCTGAATGGAG-3'
*CHS6*	5'-GAAAAGGCTCTCTTCGCAAT-3'	5'-TTCCAATTCCTCAACGTTCC-3'
*CHS3*	5'-GGCGACGTTTTGGATTTAGA-3'	5'-GCCCATTTGACAAAACCAAC-3'
*PIR3*	5'-CTCTATCGTTGCCAACAGACAGTT-3'	5'-CCAACCAGCAGCATAGATAGCA-3'
*CHS4*	5'-GCTACTCATCCGGAGCATTT-3'	5'-TGCGATGCTCTGTAAGCACT-3'
*KTR2*	5'-TGGTAGAACGCAATACGC-3'	5'-TGACTTTGACCCACCGTA-3'
*PST1*	5'-TCAAGGCTGCTGCTTCT-3'	5'-TGATTGCCGTCAAAAGAC-3'
*YPS4*	5'-AGTACGAGGGCCAACTGT-3'	5'-GTGTCAATGTGATGTTG-3'
*PRT1*	5'-CTCAATGAACGATGCTAAAAA -3'	5'- CTGAATTCGGTGTCAAAGTC-3'
*TIF2*	5'-TTTCACCTTACTTCCACCAA-3'	5'-ACCTTCCAAAGTCAATTCATC-3'
*TIF5*	5'-TTCCAAGAAGAAGAAGAAAGC-3'	5'-CTTCGTCATGATGTTGTGG-3'
*TIF11*	5'-AAGGAAGAAGGCCAAGAATA-3'	5'-GTCCCATCCAGACTTTCTTT-3'
*GCD7*	5'-GTTACAGAGGGGTTCCCTAA-3'	5'-GCTTTAGTGCCGATAATAACC-3'
*GCD11*	5'-TGGTGTTTTCAAATTAGGTGA-3'	5'-AGCAAACTTCAAGTCATTTTGT-3'
*ACT1*	5'-GCCATTTTGAGAATCGATTTG-3'	5'- GAAGTCCAAGGCGACGTAAC-3'

### Sucrose Density Gradients

Wild type and *myo1Δ *strains were grown overnight at 26°C to an optical density between 0.5-0.8 (OD_600_) in 200 ml complete synthetic media (CSM, 2% glucose and 1× nitrogen base). At the time of harvest, 1 ml of 5 mg/ml cycloheximide was added per 50 ml of yeast culture, then cultures were chilled on ice for 5 min. Pelleted cells were resuspended in 2.5 ml of lysis buffer (20 mM Tris-HCl, pH 8, 140 mM KCl, 5 mM MgCl_2_, 0.5 mM DTT, 50 μg/ml cycloheximide and 0.5 mg/ml Heparin). Cells were disrupted by vortexing for 20 s with 30 s intervals on ice (10 times). After cell lysis, glass beads and excess cell debris was removed by centrifugation at 2,061 × g for 5 min. at 4°C. The supernatant was transferred to a 1.5 ml microcentrifuge tube and centrifuged at 8,072 × g for 5 min at 4°C. RNA content in the resulting supernatant was determined by ultraviolet light absorbance at 260 nanometers wavelength (A_260_). 60 A_260 _absorbance units were layered onto 11 ml, 10% to 50% sucrose density gradients. The gradients were sedimented via centrifugation at 100,000 × g for 160 min at 4°C in a Beckman SW41 rotor. Forty-five fractions of 250 μl were collected from top to bottom of each gradient and A_260 _was measured. Consecutive fractions were pooled, generating a total of nine fractions for each gradient. Guanidine thiocyanate buffer containing 10% mercaptoethanol was added to each fraction and RNA extraction was performed as previously described. Extracts prepared from the wild type strain were employed to establish the normal profile for these culture conditions. The profiles included peaks with densities corresponding to 40 S and 60 S subunits preceding the monosomes (80 S fraction), and the polysomes were indicated. The agarose gels were included below each density profile to illustrate the electropherograms of rRNA derived from each pooled gradient fraction and their lane numbers correspond to each of the numbered fractions identified in the sucrose density gradient profiles.

### RT- PCR Analysis

For RT-PCR analysis, first-strand cDNA was synthesized from 25 ng of RNA in a total volume of 10 μl. The procedure was performed using the Quantitec Reverse Transcription kit (Qiagen, Valencia, CA) following the manufacturer's instructions. Reverse transcriptase reactions were carried out for 15 min at 42°C followed by heat inactivation for 3 min at 95°C. For each PCR reaction 3 μl of the cDNA reaction mixture together with 25 mM MgCl_2_, 10 mM dNTP, 5× FlexiGo Taq Buffer, 10 mM PCR-specific primers (Table 2) and 5 units Taq polymerase were used. The amplification reactions were performed for 30 cycles (each cycle consisted of 1 min at 94°C, 1 min at 55.7°C, and 1 min at 72 °C). Real-time RT-PCR experiments were performed in triplicate for microarray validations of a subset of genes and the quantitative data was obtained directly from the instrument (BioRad iCycler) (Table 3 and 4). Semi-quantitative RT-PCR experiments for validation of regulated mRNAs from polysome gradient fractions were performed in duplicate. The RT-PCR products were examined by 2% agarose gel electrophoresis and scanning densitometry. Quantification of mRNAs from RT-PCR experiments was determined by dividing the value of pixels/square area (mm) from the experimental mRNA RT-PCR product by the value of pixels/square area (mm) generated by *ACT1 *mRNA RT-PCR products from the same sample and averaging the results of two experiments. The Standard Error of the Mean (S.E.M.) was calculated for each experiment and results were expressed in histogram form with each set of gel images.

**Table 3:** Confirmation of microarray data by real time RT-PCR assay on a selected set of mRNAs from myo1Δ strains.

Gene	Fold Change in Microarray (p ≤ 0.01)	Fold Change by Real Time RT-PCR (± s.d.)
*RPS8A*	-7.5	-8.0 ± 0.35
*RPL7B*	-1.6	-3.6 ± 0.20
*RPL3*	-2.5	-2.3 ± 0.33
*CHS3*	-1.9	-1.3 ± 0.21
*CHS6*	1.3	1.4 ± 0.10
*YPS4*	1.6	1.5 ± 0.33
*KTR2*	2.7	1.9 ± 0.12
*PST1*	5.0	3.9 ± 0.19
*PIR3*	15.0	2.3 ± 0.18

**Table 4:** Translation initiation factors down regulated in myo1Δ strains.

Initiation Factor/Gene Names	**Function **(http://www.yeastgenome.org)	Fold Change in Microarray (p≤0.01)	Fold Change by Real Time RT-PCR (± s.d.)
eIF1Ad (*TIF11)*	Forms a complex with Sui1p (eIF1) and the 40 S ribosomal subunit and scans for the start codon	-2.41	-2.52 ± 0.42
eIF2γ (*GCD11*)	Identification of the start codon	-1.43	-1.26 ± 0.21
eIF2Bβ (*GCD7*)	Guanine-nucleotide exchange factor for eIF2	-1.29	-1.38 ± 0.27
eIF5 (*TIF5*)	GTPase-activating protein mediates hydrolysis of ribosome-bound GTP	-1.25	-1.60 ± 0.47
eIF4A (*TIF2*)	RNA helicase	-1.32	-2.14 ± 0.50
eIF3β (*PRT1*)	Binding of mRNA and Met- tRNA_i _to ribosomes	-1.39	-1.87 ± 0.10

### Western Blot Analysis

Cells were centrifuged for 5 minutes at 2,061 × g, washed in ice cold CSM media and resuspended in lysis buffer (50 mM Tris-HCl, pH 7.5, 10% glycerol, 1% Triton × 100, 0.1% SDS, 150 mM NaCl, 5 mM EDTA, 5× Protease Inhibitor Cocktail (50× stock; Roche), 10 mM PMSF). Cells were disrupted by vortex mixing for 20 seconds with 30-second intervals on ice (repeated 10 times). Protein extract was centrifuged at 15,115 × g for 10 minutes at 4°C, the supernatant was removed and quantified using the DC Protein Assay method (Bio-Rad, Hercules, CA). Total protein extracts (75 μg) were separated in a 10% SDS-PAGE gel and transferred to a nitrocellulose membrane at 0.37A for 1 h in a Mini Trans Blot Cell (Bio-Rad, Hercules, CA) at 4°C. For analysis of phosphorylated eIF2α, the membrane was incubated with anti-phospho-eIF2α polyclonal antibody (1:1000) in TBS (Tris Buffered Saline, Sigma Aldrich) containing 0.1% Tween 20 5% BSA (Bovine Serum Albumin, Sigma Aldrich) at 4°C overnight and rinsed in TBS 0.1% Tween 20 three times at 10 minutes per wash (Invitrogen, Camarillo, CA). Membranes were stripped and reprobed with a rabbit polyclonal antibody that recognized both the phosphorylated and unphosphorylated forms of eIF2αp from yeast (a generous gift by Dr. Thomas E. Dever). Finally, membranes were probed with monoclonal antibody against Pgk1p (1:125 dilution) (Invitrogen, Camarillo, CA) as a loading control. For eIF4Gp and eIF4Ep, membranes were incubated with a rabbit polyclonal anti-eIF4Gp (1:1000) and anti- eIF4Ep (1:500) (kindly provided by Dr. Peter Reid). Dcp2p-mCh was detected using 1:1000 anti-DsRed (CloneTech, Palo Alto, CA) polyclonal antibodies. After binding of the primary antibody, the washed membranes were incubated with a secondary antibody conjugated to HRP at a 1:5000 dilution for 1 hour at room temperature and washed again as described, developed with a chemiluminescent substrate (Super Signal West Pico, Thermo Scientific), and exposed to X-ray film at multiple exposure times. X-ray films were scanned with a Molecular Imager FX Pro Plus (BioRad) and digital image intensities were quantified using Quantity One 4.5.2 software (BioRad). The values derived from the ratio of the intensity of the test protein band relative to the intensity of its PGK loading control were averaged from duplicate experiments.

### eIF2α-P and Dcp2p-mCh immunoprecipitation

For immunoprecipitation experiments, 3 μg of total protein extracts were incubated with anti-DsRed, an anti-mCherry antibody (CloneTech, Palo Alto, CA), or anti-phospho-eIF2α (eIF2α-P) (Invitrogen, Camarillo, CA,) polyclonal antibodies (1:100) and Protein A immunobeads overnight at 4°C. The beads were washed four times with wash buffers (Buffer A1, 50 mM Tris-HCl pH 7.5,1 mM EDTA, 1% NP-40, 150 mM NaCl; Buffer A2, 50 mM Tris-HCl pH 7.5,1% NP-40, 150 mM NaCl; and Buffer A3, 50 mM Tris-HCl pH 7.5, 1% Np-40) and samples were divided into two aliquots. For protein analysis, IP supernatant was analyzed by 8% SDS-PAGE and Western blot analysis was performed as previously described. For RNA isolation, the antibody-reacted Protein A immunobeads were resuspended in 200 μl of RLT buffer. RNA was extracted using the RNeasy Mini Kit (Qiagen) for isolation of total RNA. Typically, a yield of 0.1 μg of RNA was obtained from an initial input of 3 μg of total cellular protein extract. RT-PCR analyses were performed from duplicate immunoprecipitation experiments using specific primers for *RPS8A, RPL7B, RPL3, CHS4, PIR3 *and *ACT1 *mRNAs as previously described. Positive control experiments were conducted with wild-type strains using equivalent amounts of isolated mRNA extracted from eIF4Ep immunoprecipitated fractions. These experiments yielded positive RT-PCR signals for the *RPS8A, RPL7B, RPL3, CHS4, PIR3 *and *ACT1 *mRNAs tested [Additional file 2], thereby providing proof that the input mRNA extracted from the immunoprecipitated proteins in this study was not degraded. An equivalent amount (0.1 μg) of input total RNA (extracted from cell lysates prior to immunoprecipitation) was amplified by RT-PCR with each primer pair to demonstrate that positive amplification of the specific mRNAs with each primer pair was efficient and specific [Additional file 3]. Mock immunoprecipitations were performed as a negative control for both the anti-Ds-red and anti-eIF2α-P experiments using total protein extract from wild-type strains incubated with Protein A immunobeads alone followed by RT-PCR amplification of the eluted fraction to corroborate the specificity of the co-precipitated mRNAs.

## Results

### Identification of differentially translated mRNAs in *myo1Δ *strains

**Figure 1:**
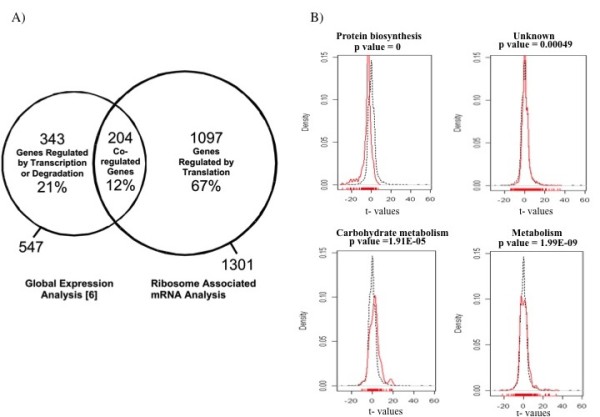
**Results of microarray analysis in *myo1Δ *strains**. A) Venn diagram illustrating the relative percentage of regulated genes. Results of global expression of mRNAs were extracted from Rodríguez-Quiñones et al. [6]. Ribosome-associated mRNAs regulated by translation were identified in the current study. Co-regulated genes are defined as differentially expressed mRNAs that were regulated in both the global analysis and ribosome-associated RNA studies. The percentage shown for each category of genes reflects the number of represented genes relative to the total number of regulated genes (1644). B) Gene Set Enrichment Analysis (GSEA) for Biological Process categories. Differentially regulated mRNAs were grouped according to Biological Process and represented graphically relative to the total population of genes in the yeast oligonucleotide microarray. Shown are histograms representing the distribution of individual genes by Density versus t-value. Only those Biological Process categories that reflected a statistically significant change in their average t-value are shown. The p-value cutoff was p≤ 0.00056. Red lines represent the distribution of genes in each specific Biological Process category. Black lines represent the distribution of all genes in the microarray. See Methods section for details.

To identify mRNAs that may be differentially regulated under stress conditions by post-transcriptional mechanisms, yeast oligonucleotide microarray studies were conducted using mRNA targets derived from ribosome fractions of wild type and *myo1Δ *strains. A total of 1,301 mRNAs were differentially bound to the *myo1Δ *ribosomes (p ≤ 0.01) [Additional file 4] of which 560 were down regulated and 741 were up regulated in the *myo1Δ *strain compared to the wild type control strains. A net 1097 mRNAs were classified as uniquely regulated at the level of translation (of which 266 changed ≥ 2-fold) (Figure 1A) representing approximately 67% of a total of 1644 regulated genes compared to 21% of the genes identified by a previous global mRNA expression analysis [6]. A total of 204 genes representing 12% of the regulated genes were co-regulated at both the translational and transcriptional levels, where the majority of the co-regulated genes were regulated in the same direction (unidirectional regulation). These co-regulated genes are identified with their corresponding GO annotations and log2-transformed fold change ratios [Additional file 5]. The GO Biological Functions included: protein biosynthesis, metabolism, stress response, cell organization and biogenesis, transport and carbohydrate metabolism among other less represented categories [Additional file 5].

To test our hypothesis that other genes of the cell wall damage transcription fingerprint [2,5] may be differentially regulated by post-transcriptional mechanisms, we checked the ribosome-associated mRNA results for representative members. Nine mRNAs of the cell wall damage fingerprint were up regulated in the ribosome pool derived from the *myo1Δ *strain, of which 4 were co-regulated at the transcriptional and post-transcriptional levels (Table 5). These were considered to be post-transcriptionally regulated cell wall stress genes. Furthermore, between the five transcriptionally up regulated genes that were previously reported and the five post-transcriptionally up regulated mRNAs identified here, we accounted for a total of 50% of the cell wall damage fingerprint (10/20) being activated in the *myo1Δ *strains. This number was below the number of genes reported to be up regulated in a *chs2Δ *mutant [7] and in a *fks1Δ *mutant (85% each) tested under identical experimental conditions [data not shown]. These observations will be discussed later.

**Table 5:** Genes representing the main transcriptional fingerprint for cell wall stress 11 that were regulated in myo1Δ strains.

Gene	Function	Fold Change (p ≤ 0.01)	
		Transcription	Translation
SLT2	Ser/Thr protein kinase	2.2	No change
FBP26	Fructose-2,6-bisphosphatase	2.2	1.9
HSP12	Heat Shock Protein	36.7	6.1
YHR097C	Unknown function	3.32	1.3
SED1	GPI-cell wall glycoprotein	3.7	2.8
KTR2	Mannosyltransferase	No change	2.71
PST1	Cell wall protein	No change	5.0
SRL3	Nucleic acid metabolic process	No change	2.14
YPS4	GPI-Aspartic protease	No change	1.64
PIR3	Cell wall glycoprotein	No change	15.2

Genes regulated by transcription were previously reported [6,7].

To analyze the global microarray data in terms of biological process categories, Gene Set Enrichment Analysis (GSEA) was performed [27]. For this analysis a gene set was created for all the 6,256 genes contained in the microarray according to their classification in 25 biological process categories referenced by Osprey network visualization software [28] [Additional file 1], considering the t-value for each gene, and then calculating the mean of the t-value of each category to determine the average fold-change for the category. The significance of differential expression was determined using the cut-off value of p ≤ 0.00056. Of the 25 categories considered for the GSEA, four categories had a corrected p-value below the cutoff (p-value≤0.00056). These categories were *protein biosynthesis*, *metabolism*, *carbohydrate metabolism *and genes of *unknown biological functions *(Figure 1B). Among the four categories identified, *protein biosynthesis *and *carbohydrate metabolism *presented the most dramatic deviation from the normal (overall) distribution of t-values.

The histogram for the *carbohydrate metabolism *category reflected a shift in their distribution towards positive t-values (Figure 1B). Among the carbohydrate metabolism genes related to cell wall biosynthesis were 7 chitin biosynthesis genes- 4 up regulated and 3 down regulated genes [Additional file 6]. Genes involved in chitin synthesis and transport *CHS5 *(1.4), *CHS6 *(1.6) and *CHS7 *(1.4), and the essential N-acetylglucosamine-phosphate mutase, *PCM1 *(1.8 fold), were up regulated. However, there were decreases in representation of mRNAs for uridine diphosphate-N-acetylglucosamine (UDP-GlcNAc) transporter *YEA4 *(-1.2 fold), chitin synthase II *CHS2 *(-2.3 fold), and activator of Chs3p *CHS4 *(-2.2 fold), enzyme components that are an integral part of chitin biosynthesis.

Genes for glucose and glycogen metabolism (which fall under the *carbohydrate metabolism *category) were also significantly activated with 11/14 co-regulated genes [Additional file 5], suggesting a vital importance for these gene functions. Among the most highly up regulated (> 5-fold) were *TPS1 *(5.3), that encodes the synthase subunit of trehalose-6-phosphate synthase/phosphatase complex, which synthesizes the storage carbohydrate trehalose; *GDB1 *(7.3), a glycogen debranching enzyme containing glucanotransferase and alpha-1,6-amyloglucosidase activities, required for glycogen degradation; *GLC3*(7.4), a glycogen branching enzyme, involved in glycogen accumulation; *UGP1*(9.1), a UDP-glucose pyrophosphorylase (UGPase) catalyses the reversible formation of UDP-Glc from glucose 1-phosphate and UTP; *GND2*(9.9), a 6-phosphogluconate dehydrogenase catalyzes an NADPH regenerating reaction in the pentose phosphate pathway; *HXK1*(14.2), a hexokinase isoenzyme 1, a cytosolic protein that catalyzes phosphorylation of glucose; and *GPH1*(18.9), a non-essential glycogen phosphorylase required for the mobilization of glycogen.

The histogram for the *protein biosynthesis *category reflected a shift from the normal distribution towards negative t-values. Out of a total of 74 genes that were identified in this category, 69 were down regulated. Among these there was a significant representation of 46 ribosomal protein (RP) genes [Additional file 4] and six translation initiation factors (Table 4). This result was predictable due to the great quantity of genes related to *protein biosynthesis *that were down regulated in the global analysis of gene expression [6].

To more precisely relate the results of this study with previous results of the ESR studies by Gasch et al. [9] and Halbeisen and Gerber [10], we compared the genes regulated by transcription and degradation [Supplementary file 1 from ref [6]], co-regulated genes [Additional file 5], and genes regulated by translation [Additional file 4]. This analysis showed that 277/315 (88%) mRNAs with log2 fold-change ratios ≥ 2 and p-values ≤ 0.01 regulated by transcription & degradation in *myo1Δ*, were also regulated in the ESR [Additional file 7] and 142/145 (98%) mRNAs with log2 fold-change ratios ≥ 2 and p-values ≤ 0.01 co-regulated genes in *myo1Δ*, were also regulated in the ESR [Additional file 8]. Of the ribosome-associated mRNAs significantly regulated (p ≤ 0.01) in the ESR reported by Halbeisen and Gerber (in Data set S3) [10] we found 313/1097 (29%) mRNAs regulated exclusively by translation in *myo1Δ *[Additional file 9]. Comparison of post-transcriptionally regulated genes related to cell wall biogenesis in *myo1Δ *to translationally regulated genes in ESR [10] identified 7 out of 34 genes in both datasets [Additional file 6].

### Validation of expression microarray results using RP mRNA polysome profiles

**Figure 2:**
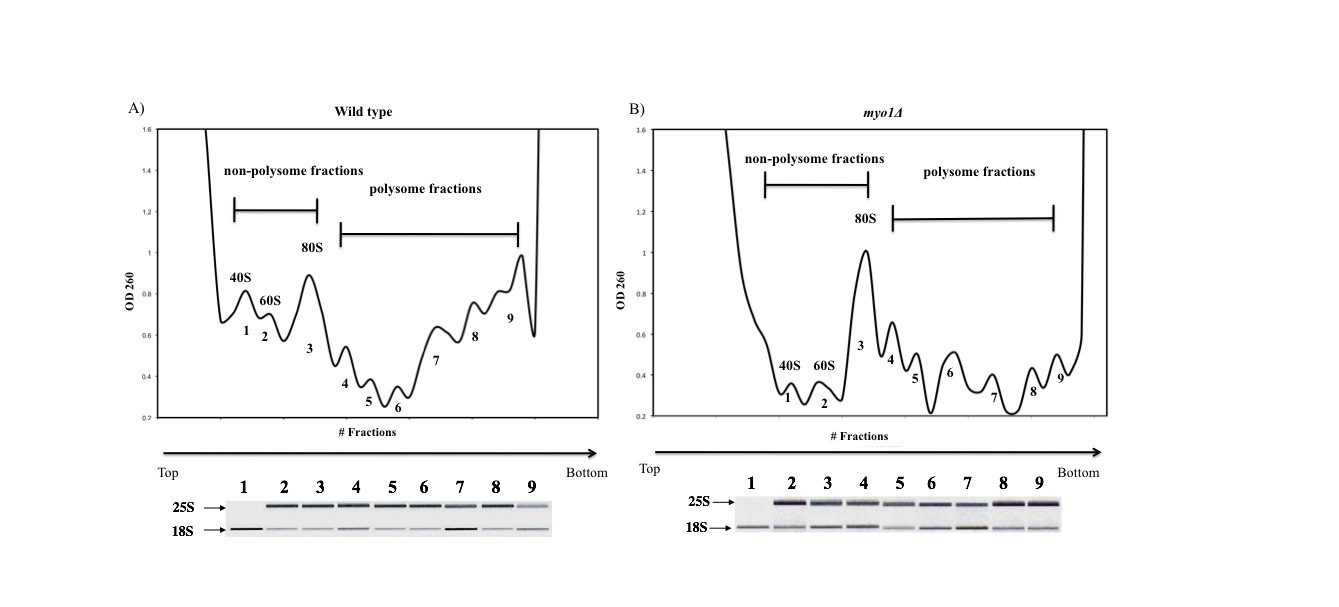
**Analysis of polysome profiles**. Equivalent amounts of total cell lysate from A) wild-type (wt) and B) *myo1Δ *strains were loaded onto 10-50% sucrose gradients. The u.v. absorbance of pooled sequential gradient fractions (numbered consecutively) was measured at 260 nm (see Methods for details). The 40 S, 60 S, and 80 S fractions were classified as non-polysome fractions. All subsequent fractions were classified as polysome fractions. Polysome peaks were numbered consecutively according to their fraction number (4-9). Total RNA extracted from each of the fractions was subjected to electrophoretic analysis of ribosomal RNAs (25 S and 18S).

To validate the interpretation of microarray data generated from translated mRNAs of *myo1Δ *strains, the overall translational activity of wild type and *myo1Δ *strains was analyzed indirectly by examining their polysome profiles using sucrose density gradients. In *myo1Δ *strains, there was a relative increase in the 80 S monosome fraction and a relative decrease in the intensity of the densest polysome peaks, fractions 7-9, indicative of a possible reduction in the efficiency of translation initiation relative to the wild type controls (Figure 2A and 2B). However, the translational repression did not apply to all genes since more than 50% of mRNAs associated with the polysomes were up regulated in the *myo1Δ *strain (Figure 1A) [Additional file 4].

**Figure 3:**
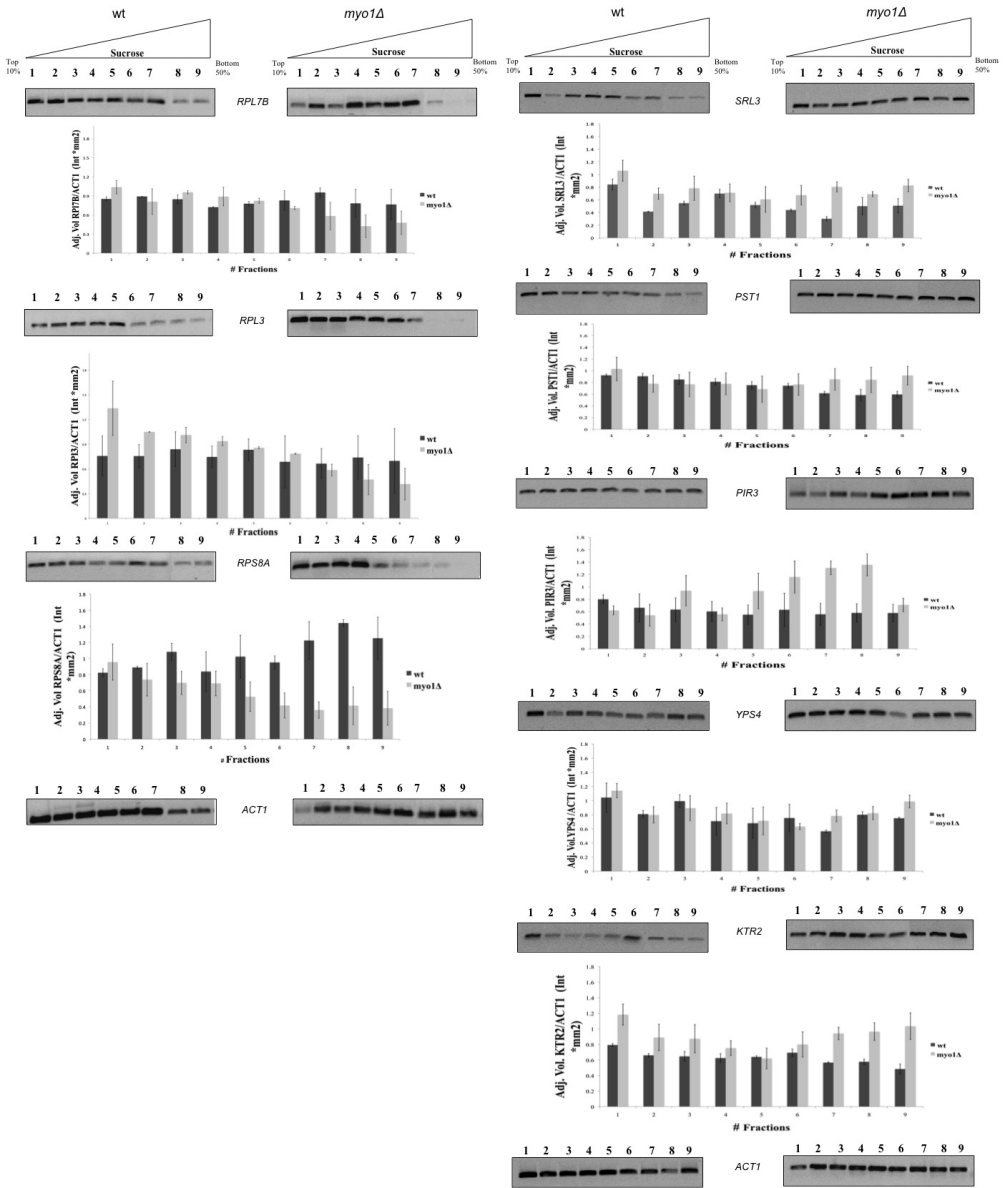
**Validation of regulated genes by RT-PCR analysis of mRNA from polysome fractions**. Total RNA was extracted from polysome fractions of wild type (wt) (left panels) and *myo1Δ *(right panels) strains. A) RT-PCR analysis of ribosomal protein mRNAs *RPL7B, RPL3, RPS8A*. B) RT-PCR analysis of mRNAs *SRL3, PST1, PIR3, YPS4*, and *KTR2 *regulated in response to cell wall damage. The RT-PCR products were resolved on 2% agarose gels, stained with ethidium bromide, and normalized against *ACT1*, a cytoskeletal protein mRNA that was not regulated in the microarray hybridization study. The lane numbers at the top of each gel correspond to the sucrose density gradient fractions shown in Figure 2. Below each set of gels is shown the results of densitometry analysis of RT-PCR products from duplicate experiments. See Methods for details.

To determine if the apparent regulation of mRNAs by translation interpreted from microarray hybridization experiments was supported by their distribution in polysome gradient fractions, three translationally regulated ribosomal protein (RP) mRNAs *RPL7B *(low-fold change sample, -1.6), *RPL3 *(medium-fold change sample, -2.9), and *RPS8A *(high-fold change sample, -8) (Figure 3A) and five translationally regulated mRNAs of the cell wall damage fingerprint *SRL3, PST1, PIR3, YPS4*, and *KTR2 *were analyzed (Figure 3B). The changes in polysome distributions were hard to differentiate in the low-fold and medium-fold change sample RP mRNAs suggesting that the polysome gradient may have limited sensitivity in these ranges. In the high-fold change sample, *RPS8A *mRNA was decreased in the heaviest polysomes fractions 6-9, and was slightly decreased in the 80 S monosomes in the *myo1Δ *strain when compared to wild type controls (Figure 3A), supporting that there was a significant decrease in the efficiency of translation of *RPS8A *mRNA. All five mRNAs representing the cell wall damage fingerprint had a greater recruitment of mRNA in the heavier polysomes (fractions 7-9), supporting that there was a relative increase in their translation efficiency, as well as in the other less dense fractions (fractions 1 and 2) in the *myo1Δ *strain (Figure 3B). Previous global mRNA expression analysis where wild-type versus *myo1Δ *strains were compared showed that none of the five translationally regulated cell wall damage mRNAs were changed at the global level (Table 5). Computer-based analysis of 5'UTR sequences of the translationally down regulated RP mRNAs [30] did not support the existence of regulatory secondary structures in their 5'UTRs as a potential mechanism for the repression of translation in this group [data not shown].

### Regulation of translation initiation factors in *myo1Δ *strains

**Figure 4:**
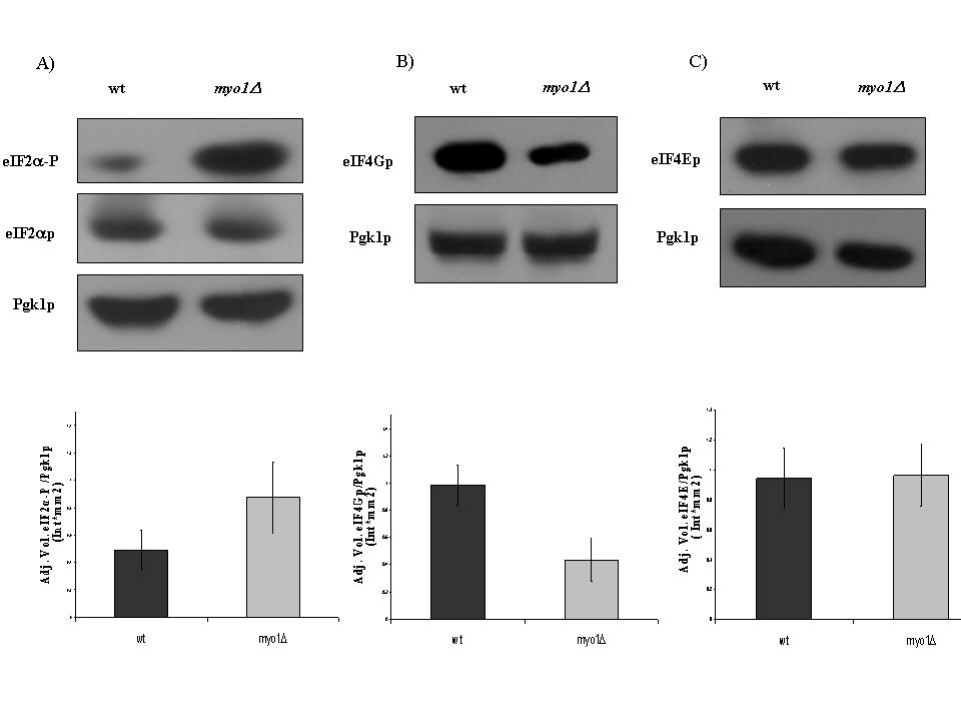
**Western blot analysis of translation initiation factors**. Equivalent amounts of total protein (75 μg) extracted from wild type (wt) and *myo1Δ *strain cultures were analyzed for the steady state levels of A) eIF2αp and it's phosphorylated form eIF2α-P, B) eIF4Gp, C) eIF4Ep. Shown are equivalent autoradiography exposures of each membrane probed with their corresponding antibodies. Pgk1p was used as a loading control in each experiment while unphosphorylated eIF2αp served as an additional loading control in panel A. The histogram below each panel illustrates the ratio of the intensity from each test protein band relative to the intensity of its PGK loading control, averaged from duplicate experiments. See Methods for details.

To establish how translation was regulated in *myo1Δ *strains, we analyzed two common mechanisms for down regulation of translation initiation described in yeast. One mechanism is the phosphorylation on Ser 51 of the alpha subunit of initiation factor eIF2p by Gcn2p protein kinase [18,19]. Phosphorylation of the alpha subunit of the initiation factor eIF2p results in reduced binding of met-tRNA_i _to the 40 S ribosomal subunit and decreased translation [18]. To test whether down regulation of translation observed in the *myo1Δ *strains was regulated by this mechanism, we analyzed eIF2p levels by Western blot analysis with antibodies specific for eIF2αp subunit phosphorylated on Ser-51 (eIF2α-P) (Figure 4A). The results show a relative increase in phosphorylation levels of eIF2αp in the *myo1Δ *strain compared to the wild type strain, while the total eIF2αp protein levels remained constant (Figure 4A). The other mechanism for down regulation of translation initiation is by the reduction in steady state levels of the initiation factor eIF4Gp [31]. When tested, the steady state levels of the initiation factor eIF4Gp were visibly decreased in *myo1Δ *relative to the wild-type strain (Figure 4B). Conversely, the steady state level of initiation factor eIF4Ep, the cap-binding component of the eIF4F complex, was not affected (Figure 4C). These experiments supported the hypothesis that translation repression was activated in the *myo1Δ *strain by Gcn2p-dependent phosphorylation of eIF2αp and by a reduction in steady state levels of eIF4Gp.

**Figure 5:**
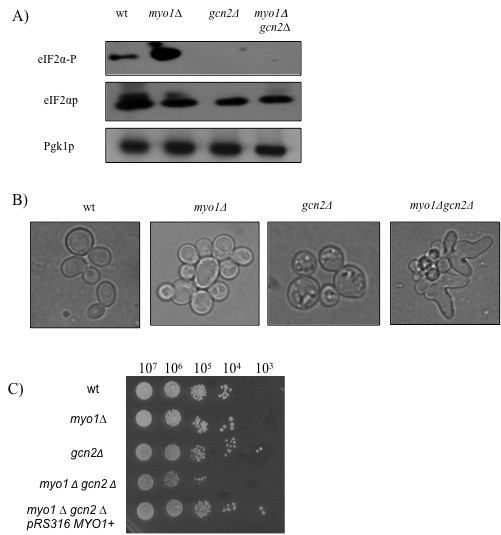
**Assessing the importance of *GCN2 *regulation**. Assays were conducted with wild type (wt), *myo1Δ, gcn2Δ *and *myo1Δgcn2Δ *strains. A) Western blot analysis of steady state levels of eIF2αp and its phosphorylated form eIF2α-P in total protein extracts derived from each strain. Pgk1p was used as a loading control, B) The morphological phenotype of each strain was observed by light microscopy at 100× magnification, C) Serial dilutions of cell suspensions from cultures of wild type (wt), *myo1Δ, gcn2Δ, myo1Δgcn2Δ*, and *myo1Δgcn2ΔpRS316-MYO1^+ ^*strains were inoculated on CSM agar medium and allowed to grow for three days at 30°C for viability assays. The strain *myo1Δgcn2ΔpRS316-MYO1^+ ^*was included to analyze complementation of the *myo1Δgcn2Δ *synthetic growth defect with a plasmid copy of the wild type *MYO1 *gene. Indicated at the top is the number of cells per 5 μL dilution.

Deletion of *GCN2 (gcn2Δ) *in both wild-type and *myo1Δ *strains abolished eIF2αp phosphorylation confirming that this regulation was mediated directly by Gcn2p (Figure 5A). In addition, a *myo1Δgcn2Δ *mutant exhibited a negative genetic interaction between these mutations characterized by an abnormal morphological phenotype (Figure 5B) and a severe growth defect (Figure 5C). The synthetic growth defect exhibited by the interaction between *myo1Δ *and *gcn2Δ *mutations was rescued by complementation with a plasmid copy of the wild-type *MYO1 *gene, *myo1Δgcn2ΔpRS316-MYO1^+ ^*(Figure 5C) indicating that it was not caused by secondary mutation(s).

### Analysis of P-bodies and identification of a novel reservoir for non-translating RP mRNAs in *myo1Δ *strains

**Figure 6:**
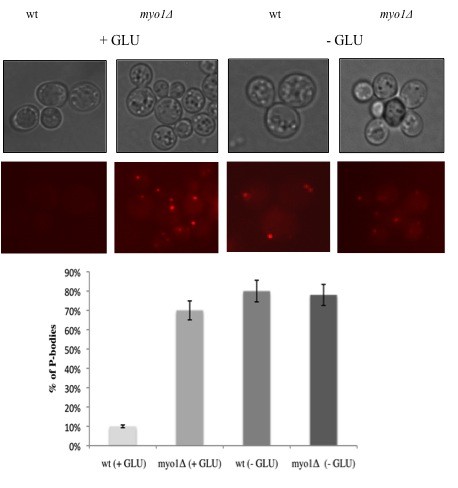
**Visualization of P-bodies *in vivo***. Wild type (wt) and *myo1Δ *yeast strains were transformed with a plasmid encoding a recombinant form of decapping enzyme Dcp2p tagged with the fluorescent probe mCherry (Dcp2p-mCh). Transformed strains were grown in CSM broth with glucose (+ GLU) and in CSM broth without glucose (-GLU). Upper panels represent light micrographs and lower panels represent the corresponding u.v. fluorescence micrographs for each condition. All images were captured at the same exposure time and magnification (100×). The graph below shows a quantification of the average number of P- bodies per 100 cells. See Methods for details.

Other laboratories have reported that non-translating mRNAs can accumulate in discrete ribonucleoprotein structures called Processing bodies or P-bodies and have correlated a reduction in their translation with an increase in the accumulation of these structures in the cytoplasm [20]. To test if P-bodies play a role in the down regulation of protein biosynthesis in *myo1Δ *strains, we used a recombinant reporter protein Dcp2p-mCherry (Dcp2p-mCh), where Dcp2p is an mRNA decapping enzyme previously reported as a component of these structures [20]. Our results indicated that 70% of *myo1Δ *cells grown in glucose-supplemented complete synthetic media accumulated P-bodies structures relative to wild type control strains in which only 10% of cells presented these structures (Figure 6 + GLU). When placed under glucose deprivation conditions, 80% of wild type cells accumulated P-bodies (Figure 6 - GLU) consistent with previous reports [20]. In *myo1Δ *cells, these structures did not increase significantly in number (78%) when subjected to glucose deprivation (Figure 6 - GLU), suggesting that the *myo1Δ *strain was subjected to a type of starved-state.

**Figure 7:**
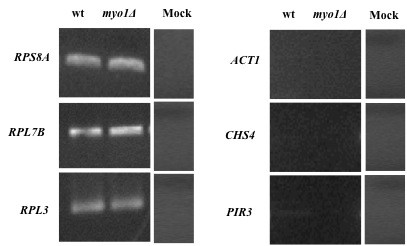
**Detection of mRNAs associated with a Dcp2p-mCh immunoprecipitated protein fraction**. Dcp2p-mCh, a protein component of P-bodies, was immunoprecipitated from whole cell protein extracts of wild type (wt) and *myo1Δ *strains. Total RNA was extracted from the immunoprecipitated fractions and RT-PCR analysis of mRNAs for *RPS8A, RPL7B, RPL3 *(left panels) and *ACT1*, *CHS4, PIR3 *(right panels) was performed. Mock immunoprecipitations were performed as a negative control for each experiment using total protein extract incubated with Protein A immunobeads alone followed by RT-PCR amplification of the eluted fraction.

Immunoprecipitation of Dcp2p-mCh protein was performed with wild type and *myo1Δ *extracts to determine if translationally repressed mRNAs were associated with this protein. Co-precipitated mRNA was extracted from immunoprecipitates and RT-PCR analysis was performed using primers for RP mRNAs *RPS8A*, *RPL3*, and *RPL7B; *and non-RP mRNAs *PIR3 *(an mRNA encoding a cell wall protein that was up regulated 15-fold), *CHS4 *(an mRNA encoding the activator of Chs3p that was down regulated 2.1-fold), and *ACT1 *(a control mRNA encoding cytoskeletal actin that was not differentially regulated by translation) (Figure 7). Results demonstrated that RP mRNAs were equally represented in the fraction containing immunoprecipitated Dcp2p-mCh protein from both the wild type and *myo1Δ *strains cultured under glucose-rich conditions (Figure 7 left panel) while *PIR3*, *CHS4 *and *ACT1 *mRNAs were absent from the same Dcp2p-mCh-positive fractions (Figure 7 right panel).

**Figure 8:**
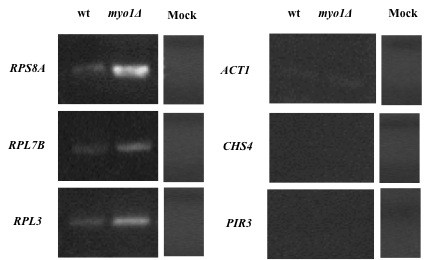
**Detection of mRNAs associated with a eIF2α-P immunoprecipitated protein fraction**. The translation initiation factor eIF2α-P was immunoprecipitated from whole cell protein extracts of wild type (wt) and *myo1Δ *strains. Total RNA was extracted from the immunoprecipitated fractions and RT-PCR analysis of mRNAs for *RPS8A, RPL7B, RPL3 *(left panels) and *ACT1*, *CHS4*, *PIR3 *(right panels) was performed. Mock immunoprecipitations were performed as a negative control for each experiment as described in Figure 7.

To investigate the selective translational repression of RP mRNAs further, we characterized their relationship with the phosphorylated form of eIF2αp. eIF2α-P was immunoprecipitated from wild type and *myo1Δ *extracts and confirmed by Western blot with eIF2α-P-specific antibody. To test if mRNA was co-precipitated with eIF2α-P, we extracted RNA from the immunoprecipitated eIF2α-P fractions and RT-PCR analysis was performed using specific primers for *RPS8A*, *RPL7B, RPL3*, *PIR3*, *CHS4*, and *ACT1 *(Figure 8). Surprisingly, we found that *RPS8A, RPL7B *and *RPL3 *were differentially co-precipitated with eIF2α-P in the *myo1Δ *strain, thereby suggesting an association between eIF2α-P-containing complexes and the non-translating RP mRNAs (Figure 8 left panel). In contrast, *PIR3, CHS4*, and *ACT1 *mRNAs were not detected in these same eIF2α-P immunoprecipitated fractions (Figure 8 right panel). To rule out mRNA degradation of *PIR3, CHS4*, and *ACT1*, an immunoprecipitation experiment was performed with eIF4E. RT-PCR of eIF4E-associated mRNA confirmed presence of these mRNAs in the translating pool [Additional file 2].

## Discussion

Eukaryotic cells have developed transcriptional and post-transcriptional controls that regulate specific cohorts of genes in response to environmental stresses. Genome-scale analysis of transcription was undertaken to identify regulated genes responding to stress conditions with the identification of repressed cohorts mostly corresponding to ribosome biogenesis and protein synthesis with activated cohorts that corresponded to energy metabolism, heat shock proteins, and other stress response genes [9]. Subsequent studies have expanded this approach to study the relation between transcriptome and translatome following transient exposure to mild and severe stress conditions resulting in the identification of characteristic coordinated responses [10]. Mild forms of stress mainly produced changes in translating mRNAs in the ribosomes with relatively minor changes in global levels of mRNAs whereas severe stress produced major changes in global transcript levels representing changes in transcription and mRNA stability, that correlated with changes in the translating mRNAs found in the ribosomes. In cells that underwent transient exposure to a severe stress, 97% of the transcriptionally regulated mRNAs were co-regulated [10]. Based on such previous studies, the expression profiles of *myo1Δ *cells resemble those generated by exposure to severe stress because co-regulation of genes was observed, although there were much fewer (only 12%) co-regulated genes. However, when we used strictly those genes that were significantly regulated 2-fold or greater for this comparison, we observed an increase in the level of co-regulation to 98% in the *myo1Δ *strain.

The predominant GO Biological Process categories represented by this group of co-regulated genes were not limited to a single category and overlapped with some of the categories previously reported by others [9,10]. A comparison of genes regulated by transcription or degradation, co-regulation, and translation levels in *myo1Δ *and ESR shows that a substantial number of genes were regulated in common. Among the Biological Process categories most represented in common between *myo1Δ *and ESR were Metabolic Process (78% of the genes in this category), Carbohydrate Metabolic Process (12%), Translation (25%), and Biosynthetic Process (48%) [Additional file 10].

Of the 20 core genes induced by different types of cell wall damage conditions, only five of these genes were up regulated globally in *myo1Δ *strains [6]. Our current analysis of post-transcriptionally regulated genes identified 9 genes, five additional genes. With the exception of *SLT2 *(not regulated post-transcriptionally) [6,7], it is not clear whether the expression of any of the remaining nine genes of the cell wall damage fingerprint is relevant to the survival of *myo1Δ *strains. A search of the *Saccharomyces *genome database (SGD, http://www.yeastgenome.org) showed that these genes share no previously reported genetic interactions with *myo1Δ *(data not shown). A rigorous test of this hypothesis will require further genetic analysis of these genes in the viable *myo1Δ *genetic background to validate any potential genetic interactions with *MYO1*. Despite the nonconformance of *myo1Δ *with full activation of the 20 reported cell wall damage genes, it was already shown that the *PKC1*-dependent cell wall integrity pathway is activated by this mutation [6,7]. Thus, we propose that impaired cytokinesis *per se *can represent another type of stress that leads to *PKC1 *activation and that *SLT2/MPK1 *regulates key cellular functions unrelated to cell wall biogenesis for survival of the *myo1Δ *strain.

The Heat Shock Protein family (*HSP*) of genes represented one of the most dramatically up regulated groups in this study. In this group we identified significant up regulation (p ≤ 0.01) of *HSP78 *(2.6 fold), *HSP42 *(5.9 fold), *HSP104 *(6.8 fold), *HSP82 *(3.0 fold), *HSP30 *(18.7 fold) and *HSP48 *(2.9 fold) [Additional file 4]. The Hsp family has a variety of functions. For example, Hsp82p/Hsp90p has been shown to play a role in regulation of protein kinase Gcn2p [32] and thus may be directly related to the putative increase in Gcn2p activity in *myo1Δ *strains. A second function of Hsp is the stabilization of other proteins to help the organism acquire tolerance to stress [33]. Lindquist and colleagues recently published that the molecular chaperone Hsp90 enables the emergence and maintenance of fungal drug resistance in *Candida albicans *[34]. The homolog of *HSP90 *in *Saccharomyces cerevisiae*, *HSP82*, was translationally up regulated 3.2 fold in the *myo1Δ *strain. However, the complex functions described for this protein preclude a converging mechanism, but the possibility of an overlapping mechanism for drug resistance and stress response is noteworthy.

The current Gene Set Enrichment Analysis resulted in two significantly regulated categories- *protein biosynthesis *and *carbohydrate metabolism*. The up regulation of genes in chitin biosynthesis supports our previous conclusion related to the importance of chitin biosynthesis for normal growth of *myo1*Δ strains [35,36]. A previous study demonstrated a synthetic lethal interaction between *chs6Δ *and *myo1Δ *[37] suggesting that correct trafficking of Chs3p was important for survival in *myo1Δ *strains. The current results expand this idea that increased chitin synthesis is due to up regulating transport and targeting of Chs3p to the plasma membrane and not the synthesis of enzymatic subunits. Such changes must be further studied at the protein level to ascertain if the observed changes in mRNA translation patterns are biologically relevant.

**Figure 9:**
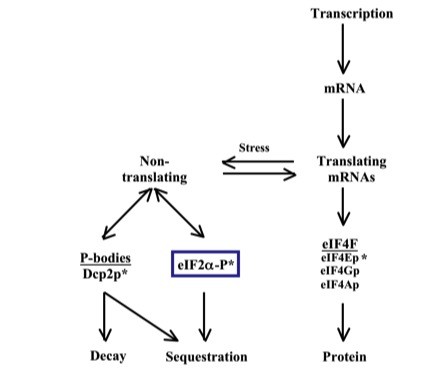
**Diagram of proposed mRNA translation control pathways**. Translating mRNAs are normally associated with eIF4F, which ultimately leads to the initiation of protein synthesis. In a process that can be mediated by stress sensors in the yeast cell plasma membrane, non-translating mRNAs may become associated with P-bodies that may lead to either mRNA decay or temporary sequestration of mRNAs for subsequent retrieval to the translating mRNA pool after adaptation to the stress. A novel eIF2α-P-containing complex is proposed as an alternative pathway for sequestration of non-translating RP mRNAs (blue-lined box). Asterisks identify immunoprecipitated proteins that were associated to translating or non-translating mRNAs in this study.

The large representation of RP mRNAs and translation initiation factors that were down regulated in the *protein biosynthesis *category, support the idea that ribosome biogenesis and translation initiation were compromised in *myo1Δ *strains. Of 137 RP genes in the yeast genome, 46 were down regulated post-transcriptionally in the *myo1Δ *strain with 83 previously shown to be down regulated by global analysis and 8 reflecting no change in their steady state or translation levels [6]. These results indicate that in addition to the reported transcriptional regulation of RP genes, there can be significant co-regulation of these genes (35/137) in *myo1Δ *strains. Furthermore, *RPS8A, RPL3 *and *RPL7B *were consistently detected in the eIF4Ep, eIF2α-P and Dcp2p-mCh-containing fractions indicating the existence of an actively translating mRNA fraction normally associated with eIF4Ep-containing complexes, a non-translating RP mRNA fraction associated with a novel eIF2α-P-containing complex, and a separate non-translating RP mRNA fraction associated with a Dcp2p-mCh-containing protein fraction (Figure 9). To our knowledge, this is the first time such a selective association between translationally repressed RP mRNAs and eIF2α-P is described. The significance of this association is yet unclear.

It is well known that the *TOR *signaling pathway is of vital importance for growth control. Certain characteristics of the *myo1Δ *strain are similar to those seen when the *TOR *signaling pathway is down regulated. For example, our microarray analysis showed that protein biosynthesis is down regulated in *myo1Δ *strains. In experiments where the *TOR *pathway has been inhibited, ribosome biogenesis has been shown to be down regulated [38]. The changes in the levels of eIF2α-P and eIF4G presented here are reminiscent of those changes associated with the inactivation of the *TOR *signaling pathway by nutrient starvation or rapamycin treatment [19,31]. Furthermore, the P-body microscopy also suggests that the *myo1Δ *strain is already in a starved-state. The dependence of eIF2αp phosphorylation on *GCN2 *expression and the *GCN2 *and *MYO1 *genetic interaction presented earlier also points to the importance of the *TOR *pathway since Gcn2p activation is regulated by the *TOR *pathway [19]. This body of evidence has prompted us to investigate whether genes of the cell wall damage fingerprint that were not regulated at the transcriptional level could be regulated post-transcriptionally via the *TOR *pathway. Western blot analysis showed that the levels of eIF2α-P and eIF4Gp in *myo1Δ *strains with or without rapamycin treatment were similar to wild-type cells treated with rapamycin (data now shown), suggesting that the *TOR *pathway was already deactivated in *myo1Δ *strains prior to treatment. A second study measuring the phosphorylation state of Npr1p (a protein kinase regulated by *TOR*) also supports that the *TOR *pathway is down regulated in *myo1Δ *strains (G. Pagán and J. Rodríguez-Medina, unpublished observation). Since inhibition of the *TOR *signaling pathway in yeast is usually associated with a nutritional deficiency, it is not clear how the *myo1Δ *strain would initiate this signal. These preliminary results nonetheless support that down regulation of the *TOR *pathway may be an important mechanism for translation repression in the *myo1Δ *condition; however, further experiments are being done to confirm this and we cannot rule out the involvement of other signaling pathways, at this point.

Initiation factor eIF4Gp serves as an anchor for the binding of other initiation factors to the preinitiation complex. Reduced steady state levels of eIF4Gp in the *myo1Δ *strains are thought to reduce the number of translation initiation events. However, eIF4Gp is also present in yeast spliceosomes and genetic depletion of Tif4631p, one of the two eIF4Gp homologues in yeast, affects the splicing of a small number of pre-mRNAs that correspond to ribosomal proteins [39]. Of the three representative RP genes tested in our study, two pre-mRNAs have introns, *RPL7B *has 2 introns, and *RPS8A *has a 5'UTR intron. If eIF4G is affecting the splicing efficiency of these RP mRNAs, a reduction in the levels of eIF4G would mean fewer fully spliced mRNAs are available for translation. Therefore, to understand the full nature of the translational repression observed among ribosomal proteins, it will be important to assess if these and other intron-containing RP mRNAs undergo eIF4Gp-dependent inhibition of splicing.

Prior studies by Texeira et al. [23] have reported that non-translating mRNAs can accumulate in discrete cytoplasmic structures called P-bodies that serve as an mRNA pool that may be translated following recovery from a given stress situation. Their results suggested additional roles for P-bodies other than for mRNA degradation, such as an inactive mRNA reservoir [20,23]. Our analysis showed that Dcp2p-mCh-positive fractions were associated with translationally down regulated *RPS8A, RPL3 *and *RPL7B *mRNAs at relatively equivalent levels in both *myo1Δ *and wild type cells despite the presence of P-bodies only in *myo1Δ *cells. However, further proof is needed to demonstrate that the Dcp2p-mCh-positive immunoprecipitated fractions actually represent P-bodies. These results do not exclude differential translational regulation of other mRNAs not tested here, by their sequestration in P-bodies.

## Conclusions

In this study, we combined microarray analysis and polysome fractionation to identify translationally regulated and co-regulated genes in *myo1Δ *cells that cannot undergo normal cytokinesis due to the absence of myosin type II [40]. Altogether, these findings indicate that yeast cells display diverse adaptive changes in gene expression regulated at both the transcriptional and post-transcriptional levels. Here we show there is a concerted post-transcriptional response to the *myo1Δ *stress with a significant cohort of co-regulated genes. We find that eIF2αp activity is down regulated in a Gcn2p-dependent manner; that Gcn2p activation implicates the *TOR *pathway; that eIF4Gp levels are down regulated; and that translation of RP mRNAs is down regulated in a manner that seems to be affected by their direct association with eIF2α-P (Figure 9). We have hypothesized that the cytokinesis defect caused by deletion of the *MYO1 *gene causes cell wall stress in addition to what may be a starved-state. Ramirez-Valle et al. reported that an energetic defect coupled to a reduction in mRNA translation rates, mimicked the defect observed by m*TOR *inhibition or nutrient deprivation in eIF4GI-silenced MCF10A cells [41]. We observed reductions in eIF4Gp levels and reductions in mRNA translation rates that could be a result of similar deficiencies in *myo1Δ *cells. Furthermore, we showed that in *myo1Δ*, regulated genes fall within functional categories of *carbohydrate metabolism *and *protein biosynthesis *described to be important for adaptation and survival in conditions of severe environmental stress [10] with some quantitative differences. In light of these previous studies, the large number of differentially up regulated genes in the *myo1Δ *strain together with the translational shutdown of others could represent a means of reprogramming gene expression to manage the chronic stress due to absence of myosin type II. In this scenario, only those mRNAs that facilitate a survival response would be efficiently translated. For example, selective translational activation of the transcription activator Gcn4p under conditions of translational repression has been previously reported in amino acid starvation conditions [42]. Alternatively, the translational down regulation of specific mRNAs encoding proteins that may be detrimental to cell survival is also possible. The line of investigation employed in this study identifies proteins involved in translationally regulated gene expression and will ultimately allow us to develop strategies to undermine the fungal stress response.

## Authors' contributions

MER-R carried out microarray experiments, ribosome fractionation, immunoprecipitation, real-time RT-PCR experiments, Western blotting, data analysis, and writing of the manuscript. JFR-Q participated in the design of microarray experiments and microarray data analysis. PA contributed to the revision and interpretation of microarray data, comparisons to the published transcriptional and post-transcriptional ESR datasets, and revision of the manuscript. JRR-M conceived the study design, coordinated its execution, carried out data analysis, wrote and revised the manuscript. All authors read and approved the final manuscript.

## Supplementary Material

Additional file 1 **Rivera-Ruiz, Rodríguez-Quiñones, Akamine, and Rodríguez-Medina**. Table of 25 biological process categories referenced by Osprey: Network Visualization System and used for GSEA in this study. Click here for file

Additional file 2 **Rivera-Ruiz, Rodríguez-Quiñones, Akamine, and Rodríguez-Medina**. Positive control experiment conducted with mRNA extracted from eIF4Ep immunoprecipitated protein fractions. Agarose gel electrophoresis of RT-PCR products is shown. Experiments yielded positive RT-PCR signals for all the mRNA primer pairs that were tested. Click here for file

Additional file 3 **Rivera-Ruiz, Rodríguez-Quiñones, Akamine, and Rodríguez-Medina**. Positive control experiment conducted with equivalent amounts (0.1 μg) of the input total RNA extracted from cell lysates prior to the immunoprecipitation step. Total RNA was amplified by RT-PCR with each primer pair and RT-PCR products were resolved on 2% agarose gels shown. The results demonstrate positive amplification for all the mRNA primer pairs that were tested. Click here for file

Additional file 4 **Rivera-Ruiz, Rodríguez-Quiñones, Akamine, and Rodríguez-Medina**. Table of post-transcriptionally regulated mRNAs (p≤0.01) in *myo1Δ *strains divided according to biological process categories. All differentially expressed genes are identified with their corresponding GO annotations, log2-transformed fold change ratios, and p-values. Click here for file

Additional file 5 **Rivera-Ruiz, Rodríguez-Quiñones, Akamine, and Rodríguez-Medina**. Table of co-regulated genes that exhibited regulation in both global expression [6] and post-transcriptional expression studies. These co-regulated genes have been identified with their corresponding GO annotations, log2-transformed fold change ratios, and p-values. Click here for file

Additional file 6 **Rivera-Ruiz, Rodríguez-Quiñones, Akamine, and Rodríguez-Medina**. Table of differentially regulated mRNAs related to cell wall biogenesis in *myo1Δ *strains at the post-transcriptional level with log2-transformed fold change ratios (p≤ 0.01). Click here for file

Additional file 7 **Rivera-Ruiz, Rodríguez-Quiñones, Akamine, and Rodríguez-Medina**. Table of genes regulated by transcription or degradation [from Supplementary file 1, reference 6] that were regulated ≥ 2-fold (p ≤ 0.01) in *myo1Δ *and ESR [9]. Click here for file

Additional file 8 **Rivera-Ruiz, Rodríguez-Quiñones, Akamine, and Rodríguez-Medina**. Table of co-regulated genes derived from Supplementary Table S2 that were regulated ≥ 2-fold (p ≤ 0.01) in *myo1Δ *and ESR [9]. Click here for file

Additional file 9 **Rivera-Ruiz, Rodríguez-Quiñones, Akamine, and Rodríguez-Medina**. Significantly regulated ribosome-associated mRNAs (p ≤ 0.01) regulated exclusively by translation in *myo1Δ *and ESR [10]. Click here for file

Additional file 10 **Rivera-Ruiz, Rodríguez-Quiñones, Akamine, and Rodríguez-Medina**. Table of genes regulated by transcription or degradation ≥ 2-fold (p ≤ 0.01) in *myo1Δ *and ESR [9] sorted according to biological process and cluster frequency. Click here for file
