# Supplementary material for: Post-transcriptional regulation in the myo1Δ mutant of Saccharomyces cerevisiae
Source: BMC Genomics. 2010 Dec 2;11:690. doi: 10.1186/1471-2164-11-690 (PMC3017085; doi:10.1186/1471-2164-11-690)
Supplement: Additional file 2 — Rivera-Ruiz, Rodríguez-Quiñones, Akamine, and Rodríguez-Medina. Positive control experiment conducted with mRNA extracted from eIF4Ep immunoprecipitated protein fractions. Agarose gel electrophoresis of RT-PCR products is shown. Experiments yielded positive RT-PCR signals for all the mRNA primer pairs that were tested. [file 1471-2164-11-690-S2.DOC]

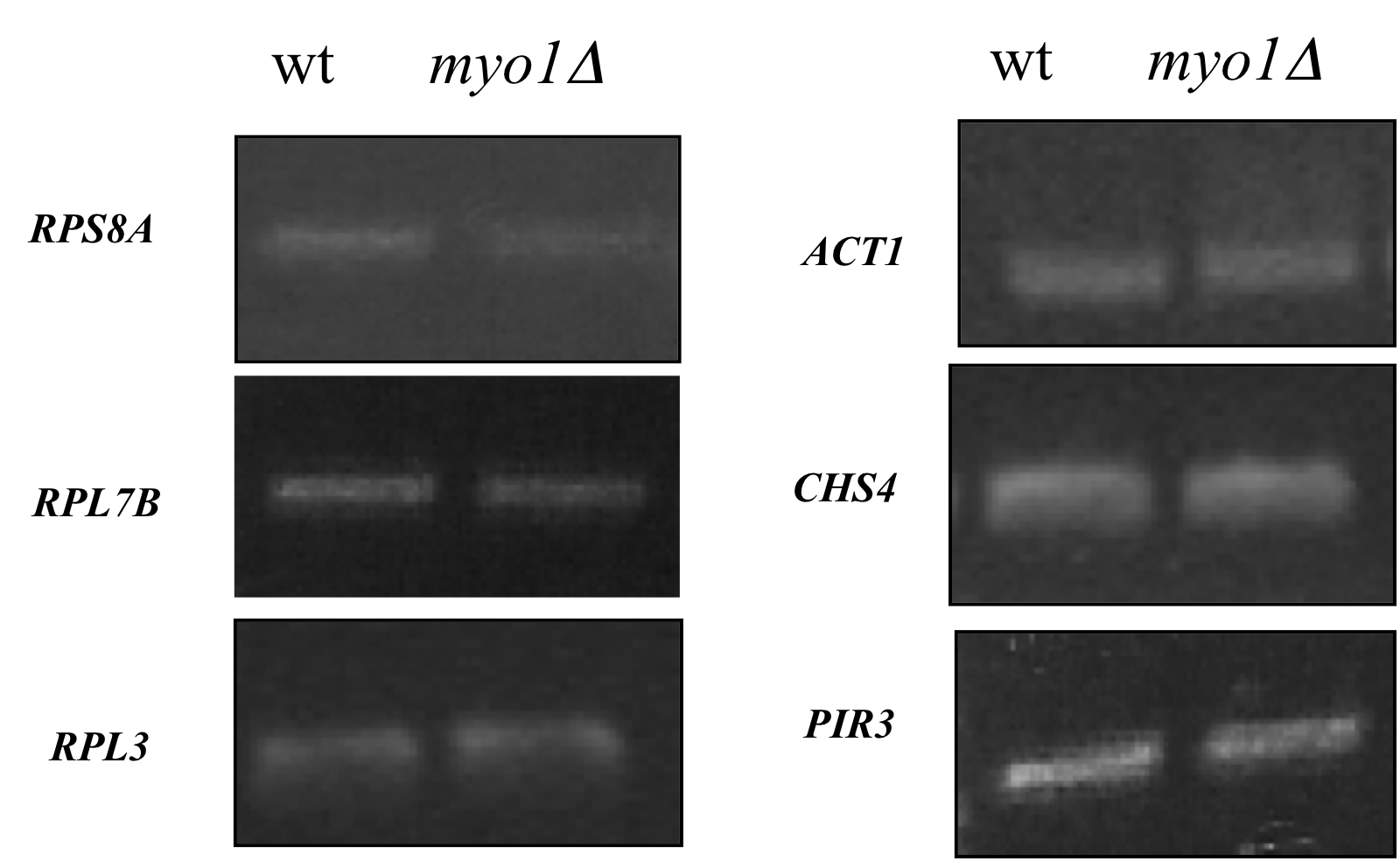


**Additional file 2**: Detection of mRNAs associated with eIF4Ep immunoprecipitated fractions. eIF4E was immunoprecipitated from whole cell extracts of wild type (wt) and *myo1Δ* strains. RNA was extracted from the immunoprecipitated fractions and RT-PCR analysis of mRNAs for *RPS8A, RPL7B, RPL3* (left panels) and *ACT1*, *CHS4,* *PIR3* (right panels) was performed.
