# Supplementary material for: Post-transcriptional regulation in the myo1Δ mutant of Saccharomyces cerevisiae
Source: BMC Genomics. 2010 Dec 2;11:690. doi: 10.1186/1471-2164-11-690 (PMC3017085; doi:10.1186/1471-2164-11-690)
Supplement: Additional file 3 — Rivera-Ruiz, Rodríguez-Quiñones, Akamine, and Rodríguez-Medina. Positive control experiment conducted with equivalent amounts (0.1 μg) of the input total RNA extracted from cell lysates prior to the immunoprecipitation step. Total RNA was amplified by RT-PCR with each primer pair and RT-PCR products were resolved on 2% agarose gels shown. The results demonstrate positive amplification for all the mRNA primer pairs that were tested. [file 1471-2164-11-690-S3.DOC]

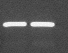

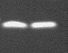

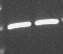

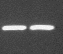

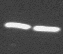


wt *myo1Δ*

*RPS8A*

*RPL7B*

*RPL3*


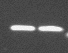


wt *myo1Δ*

*ACT1*

*CHS4*

*PIR3*

Input

**Additional file 3:** Positive control experiment conducted with equivalent amounts (0.1 μg) of the input total RNA extracted from cell lysates  prior to the immunoprecipitation step.  Total RNA was amplified by RT-PCR with each primer pair and RT-PCR products were resolved on 2% agarose gels shown.  The results demonstrate positive amplification for all the mRNA primer pairs that were tested.
